# Up-Conversion Luminescence Properties of Lanthanide-Gold Hybrid Nanoparticles as Analyzed with Discrete Dipole Approximation

**DOI:** 10.3390/nano8120989

**Published:** 2018-11-29

**Authors:** Ruichan Lv, Miao Feng, Wolfgang J. Parak

**Affiliations:** 1 Engineering Research Center of Molecular and Neuro Imaging, Ministry of Education, School of Life Science and Technology, Xidian University, Xi’an 710071, China; feng_miao@163.com; 2 Fachbereich Physik, Philipps Universitat Marburg, 35037 Marburg, Germany; wolfgang.parak@uni-hamburg.de; 3 CHyN, Universität Hamburg, 22607 Hamburg, Germany

**Keywords:** up conversion nanoparticles, discrete dipole approximation, surface plasmon resonance, lanthanide-based composites

## Abstract

Up-conversion nanoparticles (UCNP) under near-infrared (NIR) light irradiation have been well investigated in the field of bio-imaging. However, the low up-conversion luminescence (UCL) intensity limits applications. Plasmonic modulation has been proposed as an effective tool to adjust the luminescence intensity and lifetime. In this study discrete dipole approximation (DDA) was explored concerning guiding the design of UCNP@mSiO_2_-Au structures with enhanced UCL intensity. The extinction effects of gold shells could be changed by adjusting the distance between the UCNPs and the Au NPs by synthesized tunable mesoporous silica (mSiO_2_) spacers. Enhanced UCL was obtained under 808 nm irradiation. The original theoretical predictions could not be demonstrated to full extend by experimental data, indicating that better models for simulation need to take into account in homogeneities in particle morphologies. Yet, one very certain conclusion resulting from the DDA calculations and experiments is that the absorbance can blue-shift with more Au NPs added and the absorbance can-red shift for samples with enhanced silica sizes in the UCNP@mSiO_2_-Au structures. Furthermore, when the DDA model is more consistent with the practical structure (dispersed Au NPs instead of Au shell), the theoretical absorbance occurs almost the same as the practical absorbance. All in all, the DDA could fit the extinction effect of Au perfectly and be suitable for guiding how to design the UCNP and Au.

## 1. Introduction

In the context of bio-imaging lanthanide-based composites have been heavily investigated, including their synthesis, up-conversion luminescence (UCL) properties, and long-term cytotoxicity [1,2,3,4,5,6,7,8,9]. Especially, up-conversion nanoparticles (UCNPs) have been proposed as effective optical nano-probes because of the possibility to excite them in the near-infrared (NIR), which allows for deep tissue penetration, and almost negligible self-fluorescence [10,11,12,13]. In more recent years, Nd^3+^-sensitized UCL materials were proposed using 808 nm light as alternative irradiation source, which has the merit to reduce overheating of cells and to improve the penetration depth [14,15,16]. However, the low UCL efficiency is still an important problem that hinders its further application in bio-imaging [17].

Metal modulation is an important way to improve the luminescence efficiency. Metal nanoparticles, in particular gold nanoparticles (Au NPs) exhibit surface plasmon resonance (SPR) [18,19,20], i.e., when the oscillating frequency of free electrons on the metal surface is consistent with the photon frequency of the incident light, an energy resonance phenomenon occurs. The SPR effect depends on size and morphology of the Au [21,22]. Various extinction (i.e., absorption and scattering) properties of Au NPs have been used in different applications, such as scattering, dark field, and two-photon based optical imaging [23,24,25]. When Au is combined with up-conversion luminescent NPs, the scattering and absorption in the crossing visible area impairs the luminescence emission. It is proposed that all UCL peaks could be enhanced if the SPR peak would be tuned to the NIR region [26,27].

A theory to guide the design of such UCNP@Au structures would be helpful to achieve higher UCL intensities. In a previous study, we found that discrete dipole approximation (DDA) could be well used to simulate the SPR effect of gold NPs, and that there is a good agreement with measured optical properties [28,29]. On basis of this, in the present study we expand the DDA method to calculate the optical properties of lanthanide-metal nanostructures. Note that DDA permits calculation for arbitrary shapes. Although there were literatures about the combination of UCNP and SiO_2_ using the plasmonic enhancement of the gold nanoparticles [30,31], there was still no literature using DDA to guide the design of UCNP@mSiO_2_@Au composites’ structure. It is significant if the DDA simulation results could be well coincident with the real detected data. Even though there would be some disagreement between the simulation and the experimental, the simulation still has a meaningful inspiration to guide to synthesize the proper structure.

Herein, we use the discrete dipole approximation to guide the design of UCNP@mSiO_2_-AuNP structures for an enhanced UCL emission. The extinction effects of the gold shells were changed by adjusting the distance between the center UCNPs and surrounding Au NPs by a tunable mesoporous silica spacer. Finally, the enhanced UCL under 808 nm irradiation was experimentally verified. The present study exemplifies the employment of theoretical approaches (i.e., DDA) to describe the optical properties of gold and up-conversion luminescent NPs, in order to achieve a better understanding of the behavior of lanthanide-metal hybrid NPs.

## 2. Experimental Section

### 2.1. Reagents and Materials

Yttrium oxide (Y_2_O_3_, 99.99%), ytterbium oxide (Yb_2_O_3_, 99.9%), neodymium oxide (Nd_2_O_3_, 99.99%), yttrium chloride (YCl_3_·6H_2_O, 99.99%), ytterbium chloride (YbCl_3_·6H_2_O, 99.99%), neodymium chloride (NdCl_3_·6H_2_O, 99.9%), oleic acid (90%, tech grade), octadecene (90%, tech grade), trifluoroacetic acid (CF_3_COOH, 99%), sodium trifluoroacetate (CF_3_COONa, 98%), methanol (≥99.8%), hexane (≥98.5%), ammonia fluoride (NH_4_F, ≥99.99%), sodium hydroxide (NaOH, anhydrous, ≥97%), citric acid (H_3_Cit), chloroauric acid (HAuCl_4_), hexadecyl trimethyl ammonium bromide (CTAB, 99%), tetraethyl orthosilicate (TEOS, 99.99%), and aminopropyltriethoxysilane (APTES, molecular weight: 1800, 99%) were obtained by Shanghai Aladdin Bio-Chem Technology Co., Ltd., Shanghai, China. Ammonia water (25–28%) was purchased from Tianjin Kermel Chemical Reagent Co., Ltd., Tianjin, China. All chemical reagents were used as received without any further purification.

### 2.2. Synthesis of NaYF_4_:18%Yb,2%Er

The synthesis process was carried out following a previously published protocol [2]. Note that all of the synthesis was under argon atmosphere. Typically, 0.8/0.18/0.02 mmol of YCl_3_/YbCl_3_/ErCl_3_, 10 mL of oleic acid, and 15 mL of octadecene were mixed in a three-necked bottle. The mixture was heated to 160 °C and kept for 1 h to obtain a clear yellow solution (Solution A). 4 mmol NH_4_F, 2.5 mmol of NaOH was mixed together with 10 mL of methonal and sonicated for 10 min to obtain a clear solution (Solution B). Solution B was injected into the three-neck bottle directly after Solution A cooled down, and stirred for another 30 min under the room temperature. Then, the mixed solution was heated and kept at 100 °C for 20 min to remove the methanol. After that, the solution was heated to 300 °C and kept for 1 h. Finally, the solution was washed and centrifuged with ethanol to obtain NaYF_4_:18%Yb,2%Er NPs. The NP precipitate was finally dissolved in hexane.

### 2.3. Synthesis of Core/Shell NaYF_4_:18%Yb,2%Er@NaYF_4_:30%Yb,10%Nd

Following the previously published protocol [2], 0.6 mmol of Y_2_O_3_, 0.3 mmol of Nd_2_O_3_, and 0.1 mmol of Yb_2_O_3_ were mixed in a three-necked bottle with 5 mL of CF_3_COOH and heated at 120 °C until lanthanide trifluoroacetic powder was precipitated in the bottom of the bottle. Then, the as-synthesized NaYF_4_:18%Yb,2%Er NPs dissolved in hexane was added into the bottle, together with 10 mL of oleic acid and 15 mL of octadecene. After further heating and stirring at 120 °C for 30 min, the clear solution was heated and kept at 310 °C for another 30 min. Finally, the solution was washed and centrifuged with ethanol and the NaYF_4_:18%Yb,2%Er@NaYF_4_:30%Yb,10%Nd NPs were dissolved in hexane for further use.

### 2.4. Synthesis of Mesoporous UCNP@mSiO_2_

Following a previously published protocol [2], the oleic acid-capped UCNPs were firstly rendered hydrophilic by using CTAB. Typically, 2 mL of NaYF_4_:18%Yb,2%Er@NaYF_4_:30%Yb,10%Nd dissolved in hexane and 0.1 g of CTAB was mixed with 10 mL ethanol and 40 mL of deionized water under continuous stirring. After stirring for 12 h, the hexane was removed and the solution turned to be clear. Then, 1 mL of pure ammonia water (25–28%) was added. The mixture was heated and kept at 70 °C, and 150 μL of TEOS was added slowly. After 10 min reaction, the mixture was centrifuged and washed with ethanol and water for several times. The samples with different silica thickness were synthesized by adjusting the amount of TEOS (0.15 mL, 0.17 mL, 0.20 mL, and 0.30 mL). To obtain the mesoporous silica shell, the CTAB surfactant should be removed further. Typically, the as-synthesized silica-coated NPs were mixed with 30 mL of ethanol with 0.3 g of NH_4_NO_3_, and then kept at 60 °C for 2 h. Then, the solution was centrifuged with ethanol three times and the NP precipitate was collected (denoted as UCNP@mSiO_2_ in the following).

### 2.5. Synthesis of Mesoporous UCNP@mSiO_2_-Au

UCNP@mSiO_2_ NPs were dispersed in 20 mL of deionized water and then mixed with 0.1 mL of APTES solution. After stirring for 2 h at 45 °C, the solution was centrifuged and the NP precipitate was washed with deionized water. 400 μL of HAuCl_4_ (1 g/L) mixed with 45 mg of sodium citrate were stirred for 20 min at room temperature in 20 mL of deionized water, and then 0.1 mol of NaBH_4_ solution (kept in ice water) were added quickly into the solution to obtain the gold NPs. Then, the sample was mixed with 4 mL of the obtained gold nanoparticles for another 2 h at room temperature.

### 2.6. Characterization

Images of the morphology of the NPs were obtained digitally with scanning electron microscopy (SEM, JSM-6480A, Xi’ an, Shaanxi, China) and transmission electron microscopy (TEM; FEI Tecnai G2 STwin, Xi’ an, Shaanxi, China). Absorbance spectra were recorded with an UV-1601 ultraviolet visible (UV-vis) spectrophotometer. UCL emission spectra were obtained on an Edinburgh FLS980 apparatus using a 980 nm laser diode module (MDL-III-980-2W) as irradiation source and recording in the visible light region.

### 2.7. DDA Simulation

The DDA calculation was carried out by DDSCAT in the Windows 10, and it starts by dividing the NaYF_4_ core@mSiO_2_ shell-Au shell into a cubic array of N-point dipoles. The refractive index dispersion of NaYF_4_@mSiO_2_-Au spheres with different visible/near-infrared wavelengths (300–1000 nm) were used (as shown in Figure S1 and Tables S1–S3). The extinction (absorbance and scattering) effect could be obtain after processed iteratively among polarizable dipoles in the cells, and detected at the wavelength of 300–1000 nm. DDA process is converged with the error tolerance less than 10^−5^ when the number of dipoles larger than one thousand in order to ensure accuracy.

## 3. Results and Discussion

**Scheme 1:**
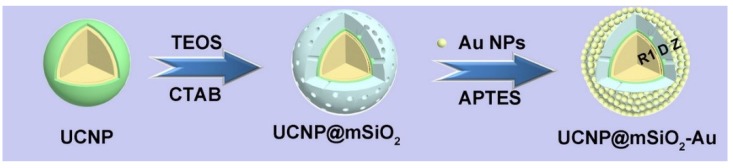
Sketch of the synthesis of UCNP@mSiO_2_-Au NPs. The radius of the UCNP core is *R*_1_, the thickness of the silica shell *D*, and the thickness of the layer of Au NPs is *Z*. In this way the radius of UCNP@mSiO_2_ is *R*_2_ = *R*_1_ + *D* and the radius of UCNP@mSiO_2_-Au is *R*_3_ = *R*_1_ + *D* + *Z*.

The schematic diagram of the synthesis process of UCNP@mSiO_2_-Au hybrid NPs is shown in Scheme 1. The structure consists of the UCNP core (radius *R*_1_), the mesoporous silica shell (thickness *D*), and then the shell of gold NPs grown on top of the mesoporous silica (thickness *Z*).

**Figure 1:**
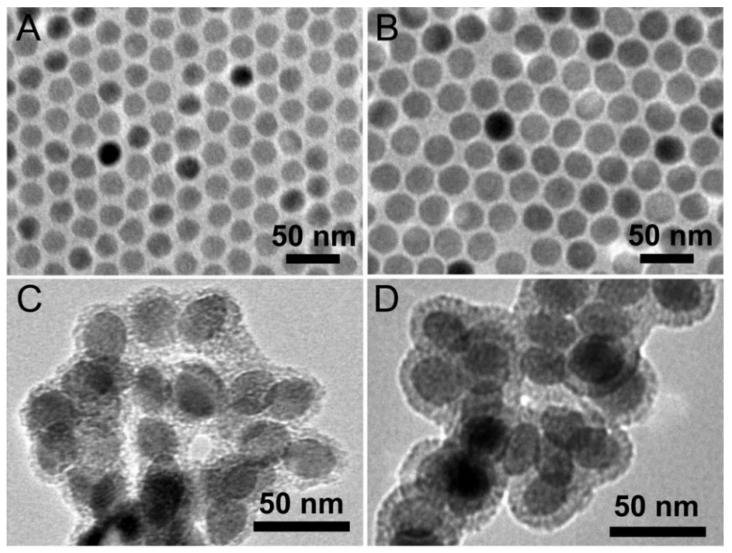

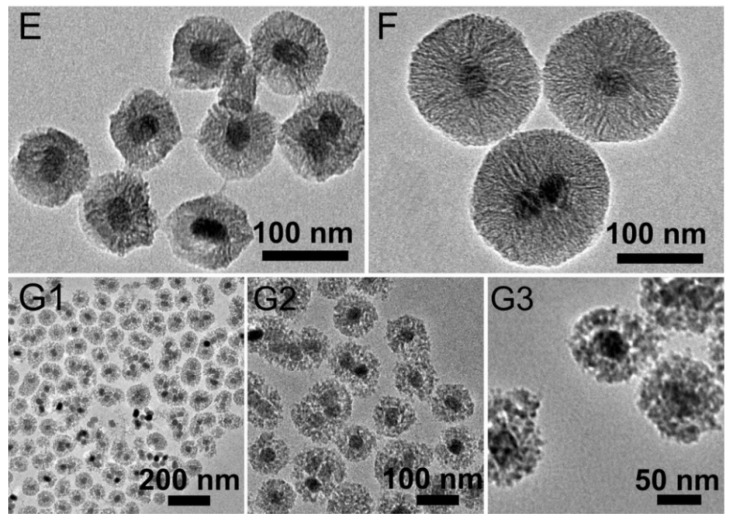
TEM images of (**A**) NaYF_4_:Yb,Er and (**B**) NaYF_4_:Yb,Er@NaYF_4_:Yb,Nd UCNPs. TEM images of UCNP@mSiO_2_ (*R*_1_ = 15 nm) with different mesoporous silica sizes: (**C**) *D* = 5 nm, (**D**) 15 nm, (**E**) 30 nm, and (**F**) 60 nm. (G1–G3) TEM images of UCNP@mSiO_2_-Au NPs (*R*_1_ = 15 nm, *D* = 30 nm, *Z* = 2–4 nm) at different magnifications.

**Figure 2:**
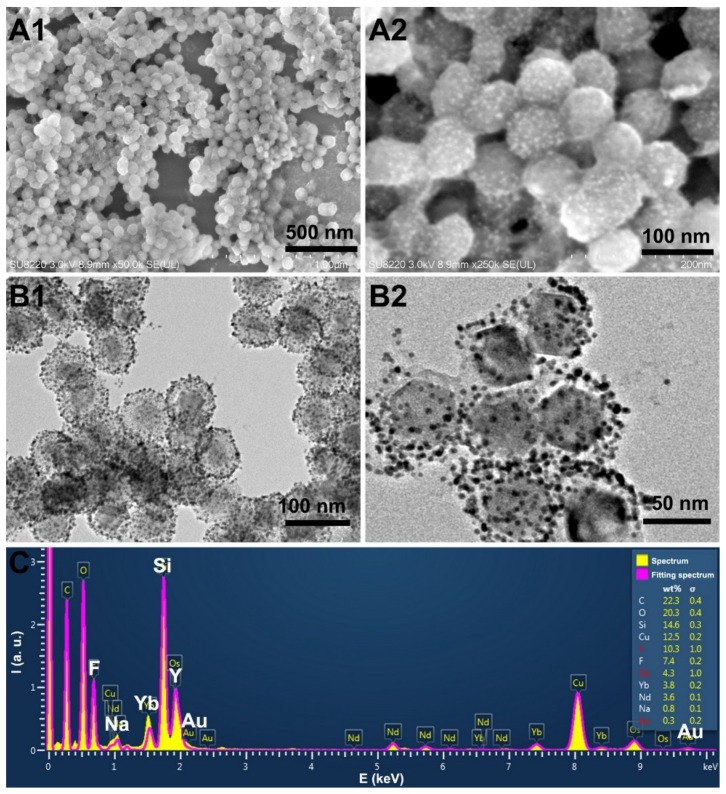
(**A1**,**A2**) SEM images at different magnifications, (**B1**,**B2**) TEM images at different magnifications, and (**C**) EDS result of UCNP@mSiO_2_-Au NPs (*R*_1_ = 15 nm, *D* = 15 nm, *Z* = 2–4 nm), in which the intensity peaks in the I(E) curve correspond to the differences in energy E between two states of the electron shell which are characteristic to the atomic structure of the emitting element.

**Figure 3:**
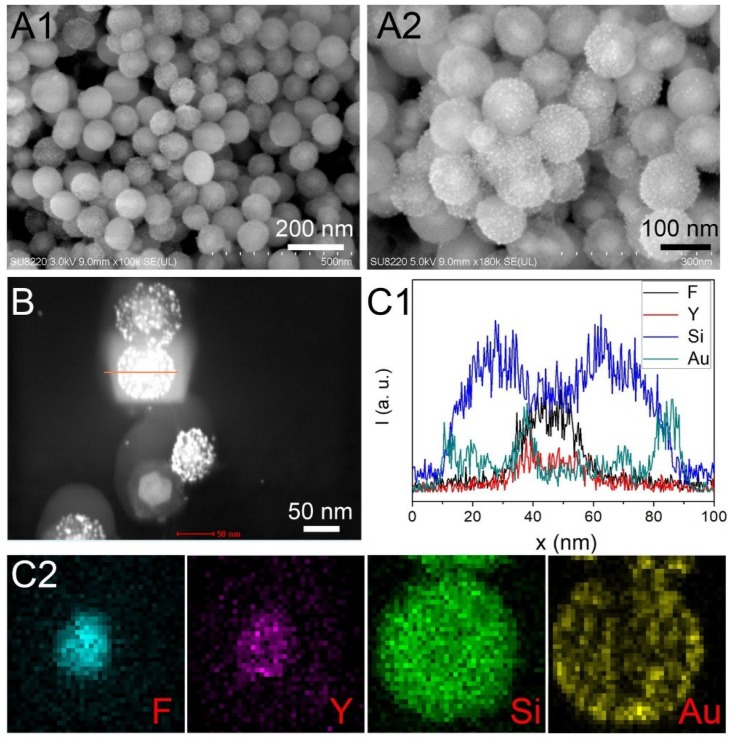
(**A1**,**A2**) SEM images at different magnifications, (**B**) High-angle annular dark-field scanning transmission electron microscopy (STEM) images, (**C1**) line scanning intensity *I*(x) with x indicating the position, and (**C2**) map scanning images of UCNP@mSiO_2_-Au NPs (*R*_1_ = 15 nm, *D* = 30 nm, *Z* = 2–4 nm).

TEM images of NaYF_4_:Yb,Er and NaYF_4_:Yb,Er@NaYF_4_:Yb,Nd UCNPs are shown in Figure 1A,B. The radius R_1_ of the NaYF_4_:Yb,Er NPs is about 11 nm. After further epitaxial growth, the radius R_1_ of the NaYF_4_:Yb,Er@NaYF_4_:Yb,Nd NPs is around 15 nm. TEM images of the as-prepared UCNP@mSiO_2_ samples with different mesoporous silica shells are shown in Figure 1C–F. Examples of shell thicknesses D with 5 nm, 15 nm, 30 nm, and 60 nm are shown. This demonstrates that the thickness of the silica shell could be well adjusted by adjusting the added amount of TEOS. In Figure 1G1–G3, images of UCNP@mSiO_2_-Au NPs (*D* = 30 nm) are shown. As shown in Figure 2A,B, the Au NPs could be clearly found on the surface and in the mesoporous shell of silica. The energy-dispersive X-ray spectroscopy (EDS) results of UCNP@mSiO_2_-Au NPs (*D* = 15 nm) are presented in Figure 2C, which show presence of Na, Y, Yb, Nd. F, Si, and Au. Furthermore, SEM images at different magnifications and high-angle annular dark-field scanning transmission electron microscopy (STEM) images of UCNP@mSiO_2_-Au NPs (*D* = 30 nm) are shown in Figure 3A1,A2,B, respectively. The line scanning intensity and map scanning images of UCNP@mSiO_2_-Au NPs (*D* = 30 nm) in Figure 3C1,3C2 indicate the distinct distribution of the UCNP core and silica mesoporous shell. The thickness Z of the layer of the Au NPs was 2–4 nm (as shown in Figure 2B and Figure 3B). The silica shell was acting as spacer between the UCNP core and the Au NP shell. The nomenclature of the NPs with different UCNP core radius *R*_1_, silica shell with thickness *D*, and Au NP shell with thickness *Z* is termed *R*_1_-*D*-*Z*.

**Figure 4:**
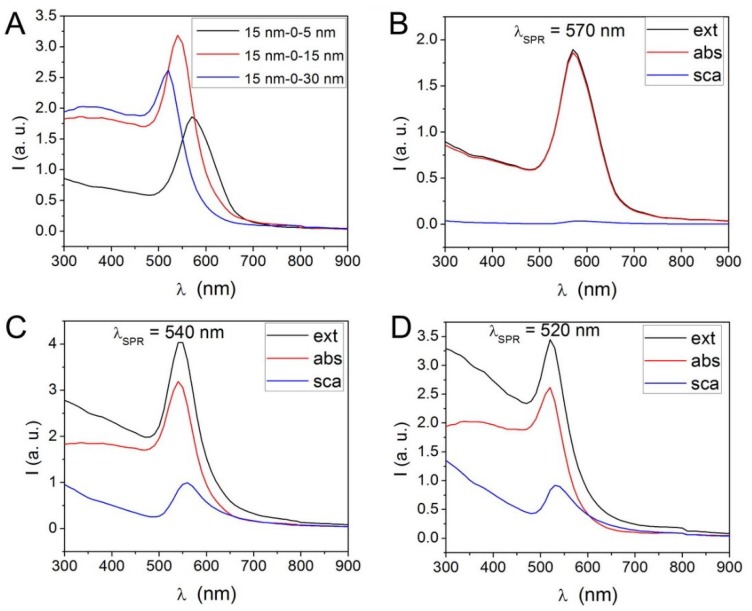
DDA simulation results *I*(*λ*), referring to the wavelength-dependence of extinction (ext), absorption (abs), and scattering (sca). (**A**) The extinction curves of UCNP-Au NPs with different Au NP shell sizes, with geometry *R*_1_-0-*Z*. The contribution of absorbance and scattering to the extinction curves is shown for (**B**) 15 nm–0–5 nm, (**C**) 15 nm–0–15 nm, and (**D**) 15 nm–0–30 nm geometry.

The DDA method was used to perform in accordance to previous studies [28,29]. The electro-dynamic simulations using the dielectric functions of the confined gold and NaYF_4_ parts were as inputs. Figure 4A shows the final extinction curves of UCNP-Au NPs (i.e., NPs without silica spacer, *D* = 0) for UCNP cores with radius *R*_1_ = 15 nm with different sizes of the Au NPs shell, as derived using DDA simulations. It is known that for nanoscale structures scattering is usually much smaller than absorption, and therefore the latter dominates the optical response. As shown in Figure 4B, for the UCNP-Au NPs with *Z* = 5 nm (i.e., the whole radius *R*_2_ = *R*_1_ + *D* + *Z* = 15 nm + 0 + 5 nm = 20 nm), the scattering is very weak and can be basically ignored. When the thickness of the Au NP shell increases to *Z* = 15 nm and 30 nm, the scattering increases (Figure 4C,D). As known that the increased scattering effect of the composite in the visible emission region would be harmful to the final UCL intensity. Also, one can see that the SPR peak of the Au NP shell is blue-shifted (from *λ*_SPR_ = 570 nm to *λ*_SPR_ = 520 nm) with increasing *Z*. Thus, a thickness of the Au NPs shell with *Z* = 5 nm is favorable and is used in the following.

**Figure 5:**
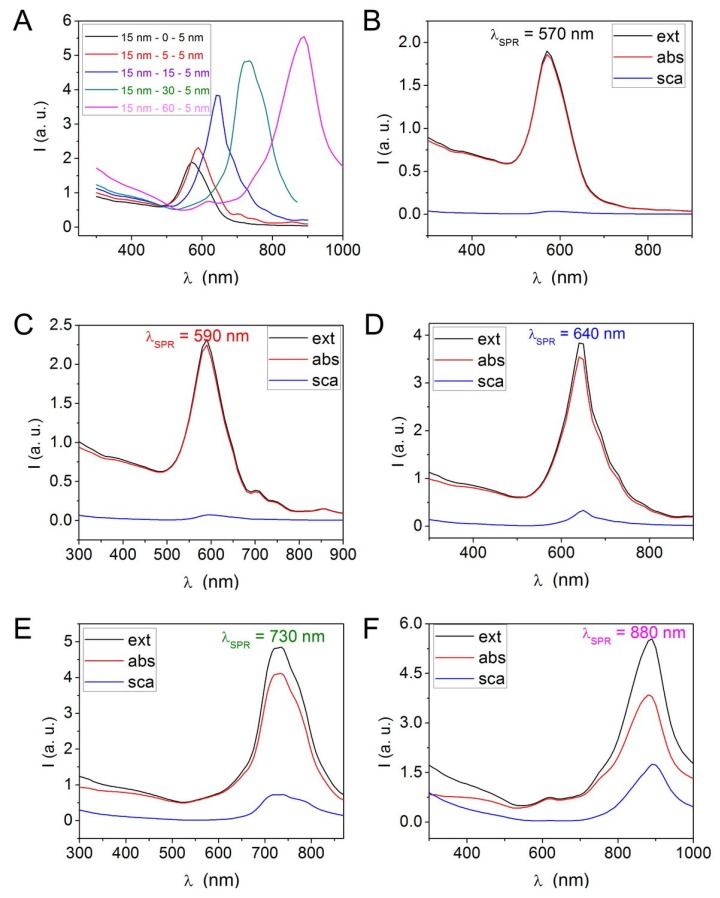
DDA simulation results *I*(*λ*), referring to the wavelength-dependence of extinction (ext), absorption (abs), and scattering (sca). (**A**) The extinction curves of UCNP@mSiO_2_-Au NPs with different silica shell thickness *D*. The extinction (absorbance and scattering) curves of UCNP@mSiO_2_-Au NPs with silica shell thickness *D* of (**B**) 0 nm, (**C**) 5 nm, (**D**) 15 nm, (**E**) 30 nm, and (**F**) 60 nm.

To obtain a hybrid structure with tunable SPR wavelength *λ*_SPR_, a spacer of mesoporous silica with adjustable distance D between the UCNP core and the Au NP shell was introduced. The extinction curves of UCNP@mSiO_2_-Au NPs with different silica shells as calculated by DDA simulation are shown in Figure 5A. There is an obvious red shift of the SPR peak when the thickness of the silica spacer is increased. The extinction (absorbance and scattering) curves of UCNP@mSiO_2_-Au NPs with different shell thicknesses of *D* = 0 nm, 5 nm, 15 nm, 30 nm, and 60 nm are presented in Figure 5B–F. The wavelengths of the corresponding SPR peaks are *λ*_SPR_ = 570 nm, 590 nm, 640 nm, 730 nm, and 880 nm, respectively. The results indicate that the adjustable silica spacer size has an obvious influence on the final optical extinction. 

**Figure 6:**
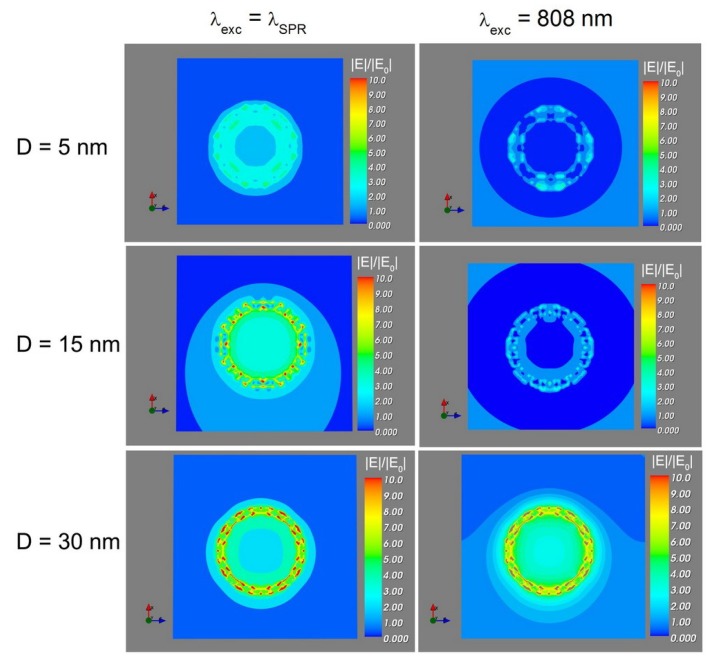
Simulation of the electric field strength (|*E*|/|*E*_0_|) of UCNP@mSiO_2_-Au NPs (15 nm–*D*–5 nm geometry) under the irradiation wavelength *λ*_exc_ = *λ*_SPR_ and *λ*_exc_ = 800 nm, with different silica spacers with thickness *D* of 5 nm, 15 nm, and 30 nm. |*E*|/|*E*_0_| is the enhancement factor. The electric field is amplified in case |*E*| > |*E*_0_|.

In order to better visualize the optical properties of the lanthanide-gold hybrid NPs, the electric field strength (|*E*|/|*E*_0_|) of different UCNP@mSiO_2_-Au NPs was calculated at excitation at the SPR resonance *λ*_exc_ = *λ*_SPR_ and at *λ*_exc_ = 808 nm, which would be the envisaged excitation in bioimaging experiments. As presented in Figure 6, when the distance between UCNP core and the gold NP shell is 30 nm, there is the strongest field strength at the excitation peak at 808 nm and SPR peak. DDA simulations thus predict 15 nm–30 nm–5 nm as the most favorable UCNP@mSiO_2_-Au NP structure.

**Figure 7:**
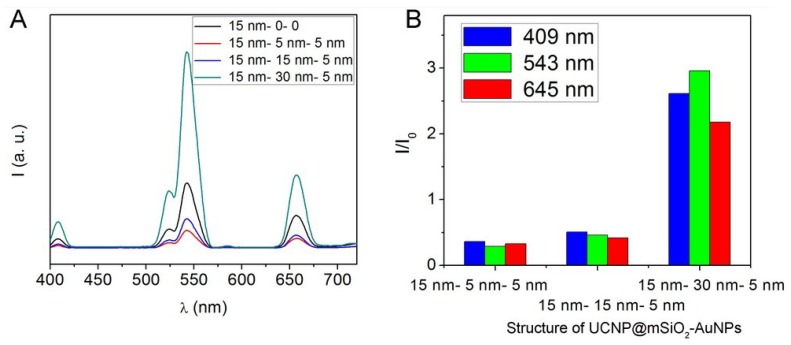
(**A**) Experimental UCL spectra *I*(*λ*) of UCNP@mSiO_2_-Au NPs with different structure and (**B**) the corresponding enhancement factor *I*/*I*_0_ corresponding to the intensity maxima at the different emission regions. *I*_0_ refers to the intensity of the plain UCNPs (geometry 15 nm–0–0).

**Figure 8:**
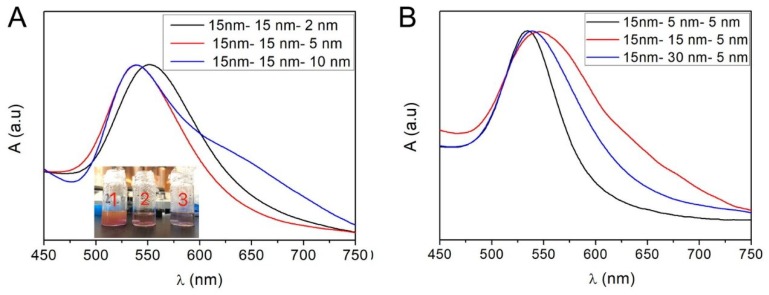
Experimental absorbance curves of UCNP@mSiO_2_-Au NPs (**A**) with different Au NPs added and (**B**) with different distance *D* between the UCNPs and Au NPs. The inset figure of Figure 8A shows the corresponding UCNP@mSiO_2_-Au solutions with enhanced Au NPs.

In order to verify the DDA predictions the UCL spectra of as-synthesized UCNP@mSiO_2_-Au NPs with different silica spacer thicknesses (cf. Figure 1) were measured. The spectra of UCNP@mSiO_2_-Au NPs contained four chief emission peaks at around 409, 521, 543, and 654 nm, corresponding to the ^2^H_9/2_ → 4I_15/2_, ^2^H_11/2_ → ^4^I_15/2_, ^4^S_3/2_ → ^4^I_15/2_, and ^4^F_9/2_ → ^4^I_15/2_ transitions of Er^3+^, respectively (Figure 7A). One can see that for a mesoporous silica spacer of thickness *D* = 30 nm, the UCL intensity at excitation *λ*_exc_ = 808 nm enhanced more than 2-fold for different emission wavelengths *λ*_em_ (Figure 7B): 2.61-fold (*λ*_em_ = 409 nm), 2.96-fold (*λ*_em_ = 543 nm), and 2.18-fold (*λ*_em_ = 645 nm), respectively. In this way the experimental data verify the predictions as achieved by DDA simulations. However, when we detected the actual absorbance properties of the solutions with different Au NPs added and with different distances between the UCNPs and Au NPs the results did not fully match the predictions (Figure 8A compared with Figure 4A, Figure 8B compared with Figure 5A).

Discrepancy in the absorption spectra may be explained by the different distribution of the Au NPs in the silica shell, which in the case of the synthesized Au NPs is dispersed. Uncertainty in the geometry of the deposited Au NPs on the surface of the mesoporous silica shell (including dispersion, number, and location) lead to inexact simulation results. First attempts to use more realistic geometries in the simulation failed so far. Still, the absorbance blue-shift of samples with more Au NPs added (Figure 8A) and the absorbance red-shift of samples with enhanced silica sizes (Figure 8B) qualitatively goes in the same direction as the simulated values (Figure 4A and Figure 5A). That means, DDA simulation needs to be refined taking better into account the distribution of the Au NPs for being better able to predict improved design of lanthanide@silica@Au NPs with optimized up-conversion luminescence intensity. 

**Figure 9:**
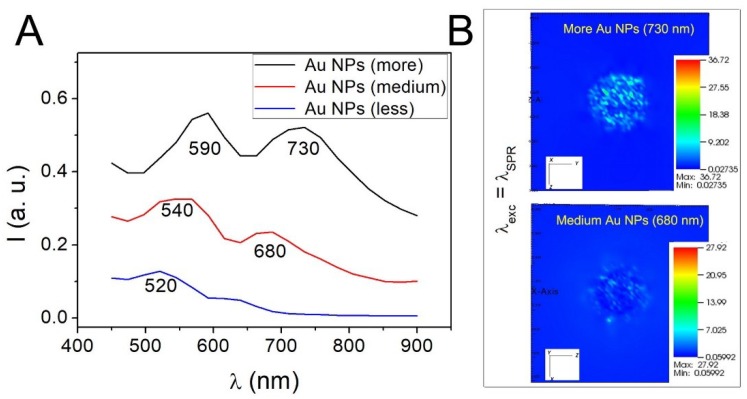
DDA results with dispersed Au NPs in the 5 nm Au shell. (**A**) The wavelength-dependence of extinction and (**B**) the electric field strength (|*E*|/|*E*_0_|) of UCNP@mSiO_2_-Au NPs (15 nm–15 nm–5 nm geometry) under the irradiation wavelength *λ*_exc_ = *λ*_SPR_.

We tried another improved DDA model with the aim to be better consistent with the experimental results. Here, a modified DDA model of UCNP@mSiO_2_-Au NPs with dispersed NPs in the shell instead of a full Au shell was carried out. As shown in Figure 9A, when there are more dispersed Au NPs in the shell, the SPR peak red-shifts in comparison to UCNP@mSiO_2_-Au NPs with less Au NPs (Note: dispersed Au NPs instead of Au shell). The electric field strength under the irradiation wavelength *λ*_exc_ = *λ*_SPR_ (730 nm and 680 nm, respectively, in case of more and medium dispersed Au NPs) further directly present higher energy in the UCNP@mSiO_2_-Au NPs with more Au NPs (Figure 9B). If the amount of Au NPs would be much lower, the NIR peak and energy may disappear, and there is only one SPR peak at 520 nm. These results may well explain why the experimental spectra has lower absorbance in the NIR region. That means, the DDA simulation could be suitable for guiding how the Au NPs effect the UCNPs if the DDA model would be improved.

## 4. Conclusions

In this work we have fabricated lanthanide@silica@Au structures with enhanced UCL intensity. DDA simulation were used to guide the design. The final UCL enhancement factor is more than 2-fold when the distance between lanthanide and Au NPs is 30 nm. The present study exemplifies the employment of theoretical approaches used to describe the optical properties to achieve better understanding of the behavior of lanthanide-metal hybrid nanoparticles, while pointing out also limitations of such modelling.

## Supplementary Materials

The following are available online at http://www.mdpi.com/2079-4991/8/12/989/s1, Table S1: The refractive index n of NaYF_4_ in dependence of the wavelength; Table S2: The refractive index n of SiO_2_; Table S3: The refractive index n of Au, Figure S1: The refractive index n (real part) of NaYF_4_, SiO_2_, and Au.
