# Supplementary material for: Up-Conversion Luminescence Properties of Lanthanide-Gold Hybrid Nanoparticles as Analyzed with Discrete Dipole Approximation
Source: Nanomaterials (Basel). 2018 Nov 29;8(12):989. doi: 10.3390/nano8120989 (PMC6315549; doi:10.3390/nano8120989)
Supplement: Supplementary file 1 [file nanomaterials-08-00989-s001.pdf]

Supporting Information

Up-Conversion Luminescence Properties of Lanthanide-Gold Hybrid Nanoparticles as Analyzed with Discrete Dipole Approximation

Ruichan Lv [1,2,*], Miao Feng [1] and Wolfgang J. Parak [2,3]

[1] Engineering Research Center of Molecular and Neuro Imaging, Ministry of Education, School of Life Science and Technology, Xidian University, Xi'an, Shanxi 710071, China

[2] Fachbereich Physik, Philipps Universitat Marburg, 35037 Marburg, Germany

[3] CHyN, Universität Hamburg, 22607 Hamburg, Germany

* Correspondence: rclv@xidian.edu.cn

Table S1. The refractive index n of $NaYF_4$ in dependence of the wavelength λ. (Re refers to the real part, and Im to the imaginary part.)

λ (nm)	Re (n)	Im (n)
450	1.497505	0
500	1.486588	0
550	1.479867	0
600	1.475527	0
650	1.472612	0
700	1.470586	0
750	1.469138	0
800	1.468075	0
850	1.467279	0
900	1.46667	0
950	1.466197	0
1000	1.465823	0

Table S2. The refractive index n of SiO_2. (Re means the real part, and Im means the imaginary part.)

λ (nm)	Re (n)	Im (n)	λ (nm)	Re (n)	Im (n)
300	1.4878	0	580	1.4587	0
305	1.4864	0	585	1.4586	0
310	1.4851	0	590	1.4584	0
315	1.4839	0	595	1.4582	0
320	1.4827	0	600	1.458	0
325	1.4816	0	605	1.4579	0
330	1.4806	0	610	1.4577	0
335	1.4796	0	615	1.4576	0
340	1.4787	0	620	1.4574	0
345	1.4778	0	625	1.4572	0
350	1.4769	0	630	1.4571	0
355	1.4761	0	635	1.457	0
360	1.4753	0	640	1.4568	0
365	1.4745	0	645	1.4567	0
370	1.4738	0	650	1.4565	0
375	1.4731	0	655	1.4564	0
380	1.4725	0	660	1.4563	0
385	1.4719	0	665	1.4561	0
390	1.4713	0	670	1.456	0
395	1.4707	0	675	1.4559	0
400	1.4701	0	680	1.4558	0
405	1.4696	0	685	1.4556	0
410	1.4691	0	690	1.4555	0
415	1.4686	0	695	1.4554	0
420	1.4681	0	700	1.4553	0
425	1.4676	0	710	1.4551	0
430	1.4672	0	720	1.4549	0
435	1.4668	0	730	1.4546	0
440	1.4663	0	740	1.4544	0
445	1.466	0	750	1.4542	0
450	1.4656	0	760	1.454	0
455	1.4652	0	770	1.4539	0
460	1.4648	0	780	1.4537	0
465	1.4645	0	790	1.4535	0
470	1.4641	0	800	1.4533	0
475	1.4638	0	810	1.4531	0
480	1.4635	0	820	1.453	0
485	1.4632	0	830	1.4528	0
490	1.4629	0	840	1.4527	0

495	1.4626	0	850	1.4525	0
500	1.4623	0	860	1.4523	0
505	1.4621	0	870	1.4522	0
510	1.4618	0	880	1.452	0
515	1.4615	0	890	1.4519	0
520	1.4613	0	900	1.4518	0
525	1.461	0	910	1.4516	0
530	1.4608	0	920	1.4515	0
535	1.4606	0	930	1.4513	0
540	1.4603	0	940	1.4512	0
545	1.4601	0	950	1.4511	0
550	1.4599	0	960	1.4509	0
555	1.4597	0	970	1.4508	0
560	1.4595	0	980	1.4507	0
565	1.4593	0	990	1.4505	0
570	1.4591	0	1000	1.4504	0
575	1.4589	0			

Table S3. The refractive index n of Au. (Re means the real part, and Im means the imaginary part.)

λ (nm)	Re (n)	Im (n)
331.5	1.48	1.883
342.5	1.48	1.871
354.2	1.5	1.866
367.9	1.48	1.895
381.5	1.46	1.933
397.4	1.47	1.952
413.3	1.46	1.958
430.5	1.45	1.948
450.9	1.38	1.914
471.4	1.31	1.849
495.9	1.04	1.833
520.9	0.62	2.081
548.6	0.43	2.455
582.1	0.29	2.863
616.8	0.21	3.272
659.5	0.14	3.697
704.5	0.13	4.103
756	0.14	4.542
821.1	0.16	5.083
892	0.17	5.663
984	0.22	6.35

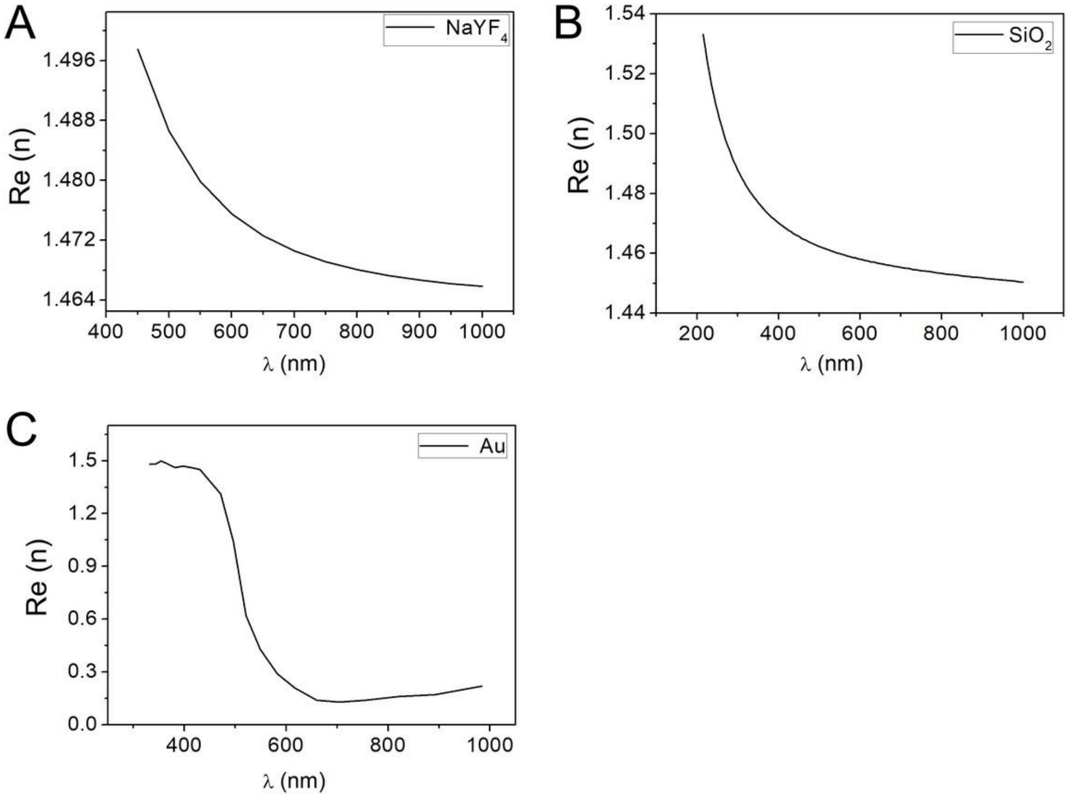

Figure S1. The refractive index n (real part) of $NaYF_4$, SiO_2, and Au.
